# Supplementary figures and images for: Exploration of Postharvest Conditions for Codonopsis pilosula Nannf. var. modesta (Nannf.) L. T. Shen Roots Based on Sensory Quality, Active Components, Antioxidant Capacity and Physiological Changes at Different Storage Temperatures
Source: Foods. 2023 Dec 9;12(24):4418. doi: 10.3390/foods12244418 (PMC10742758; doi:10.3390/foods12244418)

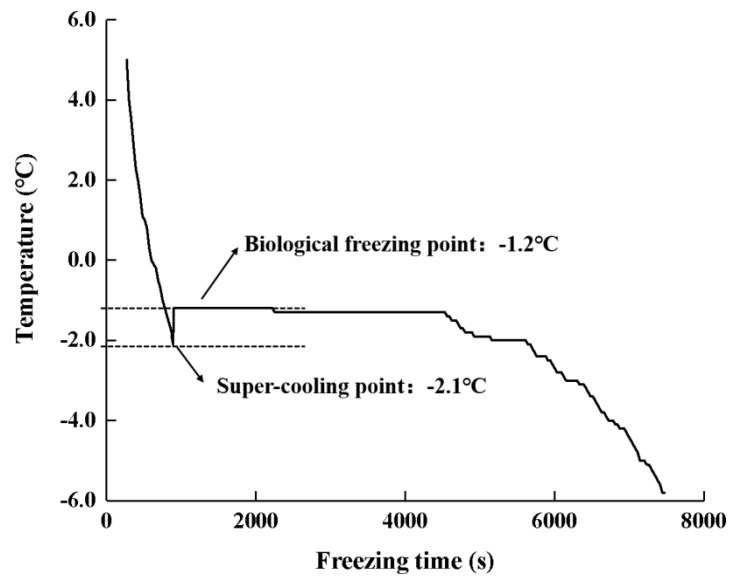

**Figure S1** Freezing point curve of postharvest *C. pilosula* roots.

Supplement: Supplementary file 1 [file foods-12-04418-s001.zip › foods-2714376-supplementary.pdf]
